# In silico Study of the Pharmacologic Properties and Cytotoxicity Pathways in Cancer Cells of Various Indolylquinone Analogues of Perezone

**DOI:** 10.3390/molecules22071060

**Published:** 2017-06-25

**Authors:** René Escobedo-González, Claudia Lucia Vargas-Requena, Edgar Moyers-Montoya, Juan Manuel Aceves-Hernández, María Inés Nicolás-Vázquez, René Miranda-Ruvalcaba

**Affiliations:** 1 Departamento de Ciencias Químicas, Facultad de Estudios Superiores Cuautitlán, Universidad Nacional Autónoma de México, Cuautitlán Izcalli, Estado de México, C.P. 54740, México; renegerardo.escobedo@gmail.com (R.E.-G.); juanmanuel.is.acevesh@gmail.com (J.M.A.-H.); mirruv@yahoo.com.mx (R.M.-R.); 2 Instituto de Ciencias Biomédicas, Universidad Autónoma de Ciudad Juárez, Henry Dunant #4600, Ciudad Juárez 32310, México; cvargas@uacj.mx; 3 Instituto de Ingeniería y tecnología, Universidad Autónoma de Ciudad Juárez, Ave. Del Charro 450 Norte, Ciudad Juárez 32310, México; edgar_moymon@hotmail.com

**Keywords:** indolylquinones, perezone, apoptosis, cancer, quantum chemistry calculations, docking study, chemioinformatics tools

## Abstract

Several indolylquinone analogues of perezone, a natural sesquiterpene quinone, were characterized in this work by theoretical methods. In addition, some physicochemical, toxicological and metabolic properties were predicted using bioinformatics software. The predicted physicochemical properties are in agreement with the solubility and cLogP values, the penetration across the cell membrane, and absorption values, as well as with a possible apoptosis-activated mechanism of cytotoxic action. The toxicological predictions suggest no mutagenic, tumorigenic or reproductive effects of the four target molecules. Complementarily, the results of a performed docking study show high scoring values and hydrogen bonding values in agreement with the cytotoxicity IC_50_ value ranking, i.e., indolylmenadione > indolylperezone > indolylplumbagine > indolylisoperezone. Consequently, it is possible to suggest an appropriate apoptotic pathway for each compound. Finally, potential metabolic pathways of the molecules were proposed.

## 1. Introduction

**Figure 1:**
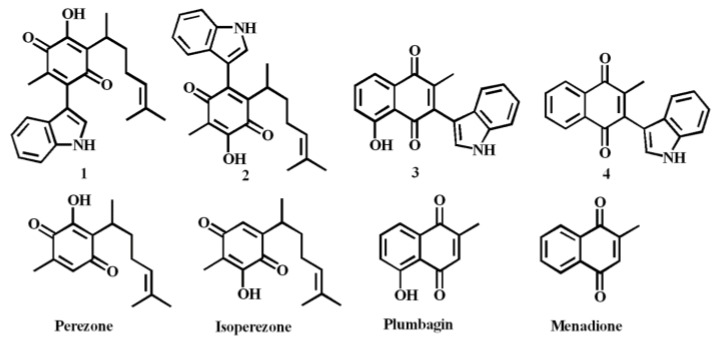
Target molecules, and their corresponding quinone raw material.

Many secondary metabolites isolated from plant specimens are quinones [1]. Perezone or pipitzaoic acid (Figure 1), obtained from specimens of the roots of *Perezia* (currently *Acourtia*) by Río de la Loza, is reportedly the first secondary metabolite isolated in the New World [2]. The molecule has been the focus of many chemical, structural and biological studies [3,4,5,6,7,8]. It has also been used in an interesting green route for the production of various derivatives [9] and many of its pharmacological effects have been also reported [10,11,12,13,14].

In recent years, cancer prevention by means of natural products has received considerable attention. Chemical modification of secondary metabolites is considered a powerful method for the construction of novel drug candidates. As the indolylquinone moiety is present in many natural products with interesting biological activities [15,16], consequently, the promising pharmaceutical use of the indolylquinones has attracted attention for the synthesis of new molecules of this class. 

As a part of our ongoing research interest in the implementation of green synthetic strategies to synergize and modify the pharmacological activities of single known compounds through the construction of novel hybrid molecules [17,18,19,20,21,22], in a recent work, we reported the cytotoxic effects on a human breast cancer cell line of a set of four indolylquinones **1**–**4** obtained from perezone, isoperezone, plumbagine and menadione, respectively [23]. Based on the corresponding cytotoxicity IC_50_ values the studied compounds could be ranked as follows: indolylisoperezone > indolylplumbagine > indolylperezone > indolylmenadione, i.e., **2** was the most active compound and **4** the least active, while **1** was less active than **3**. It should also be mentioned that the cytotoxic effect of perezone and its synthetic isomer (isoperezone) has also been studied on the K562 human leukemia cell line and perezone showed a greater cytotoxic effect than isoperezone [13].

Recently computational chemistry methods such as quantum chemical calculations, molecular modelling and chemioinformatics tools have been used in both the design of new drugs and the study of their possible mechanisms of action. Quantum-chemical calculations have been extensively used in structure-activity studies in biochemistry, where these calculations provide descriptors that can be correlated with the biological activity. Some of these descriptors are atomic charges, HOMO-LUMO energies, and frontier orbital graphs [24].

Docking studies provide models of the possible interactions of protein residues with ligands, and can also furnish potential explanations for the therapeutic action on a protein receptor. Thus, docking score values, hydrogen bonds and bonding energy values are very important in the elucidation of ligand-receptor interaction mechanisms and the possible therapeutic action of drugs under study. Binding poses with the lowest binding free energy, the number and energy of hydrogen bonds and bonding energy are selected for the optimum docking conformation [25]. Finally, the chemioinformatics tools allow an approach to certain properties of studied molecules by use of diverse methodologies applied in the drug discovery process, such as compound selection, virtual screening, and metabolic prediction [26]. Taking into account the aforementioned facts, the goal of this work was examine several physicochemical predicted properties in addition to the apoptotic pathway of four indolylquinones, by using quantum determinations, molecular docking and chemioinformatics tools, in combination with the previously reported experimental results [23].

## 2. Results and Discussion

**Figure 2:**
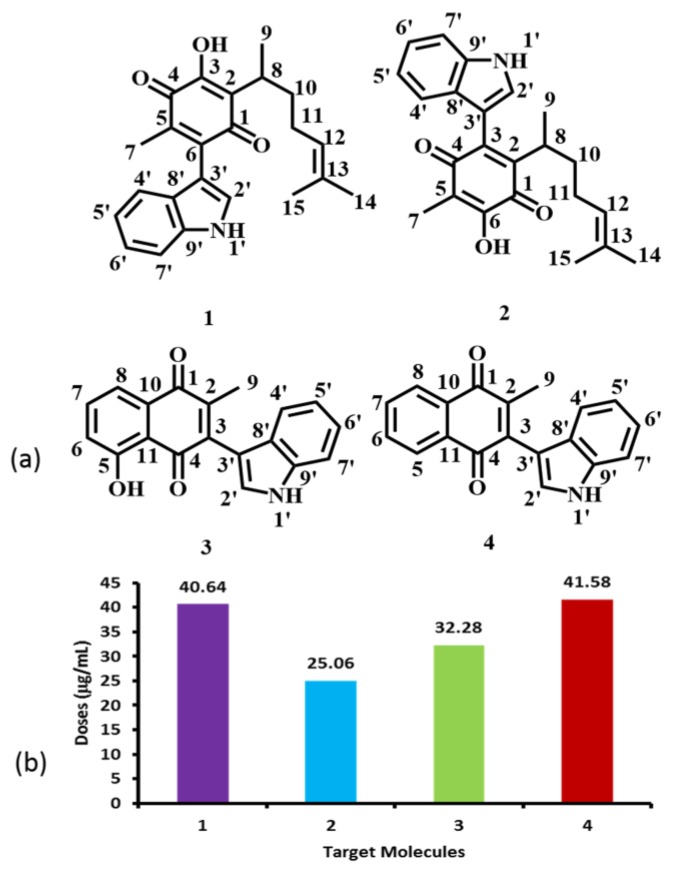
Cytotoxic effect of indolylquinones in cancer breast cells. (**a**) Atom numbering assignments of compounds **1**–**4**. (**b**) Histogram of the corresponding IC_50_ values.

As previously mentioned, our research group recently reported the cytotoxic activity of **1**–**4** (Figure 2a) in a human breast cancer cell line (MDA-MB-231) [23]. Taking into account the corresponding IC_50_ values (Figure 2b), we have now analyzed their structure-activity correlations, using the reactivity parameters calculated by both quantum chemical calculations and molecular modelling; in addition, the values of adsorption, metabolism, toxicological and physicochemical properties were predicted using chemioinformatics tools.

### 2.1. Molecular Reactivity

#### 2.1.1. Molecular Orbital Analysis

**Figure 3:**
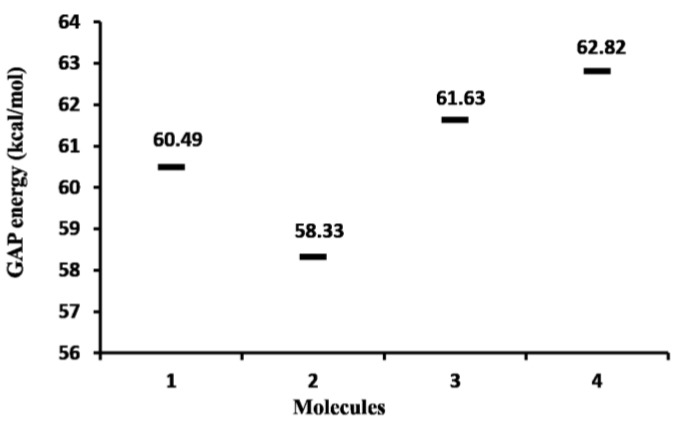
Gap energy plot (kcal/mol) of the target molecules.

Calculated gap values (E_LUMO_-E_HOMO_) of the studied molecules are displayed in Figure 3. These values show that **4**, with a ∆E_gap_ = 62.82 kcal/mol, is the least reactive (the most stable) molecule. The higher stability in **4** can be explained considering its greater aromatic nature. The most cytotoxic compound was **2** (IC_50_ = 25.06 μg/mL), which is the molecule with the smallest energy gap (E_gap_ = 58.33 kcal/mol) and largest reactivity. These results suggest a possible correlation between the chemical stability and the breast cancer cell cytotoxicity.

The value of E_HOMO_ is regularly associated with the electron donating capability, and a higher value of E_HOMO_ is indicative of a greater ease of donating electrons to unoccupied orbitals. A lower value of E_LUMO_ is related to the ability of the molecule to accept electrons. The corresponding quantum chemical parameters calculated by DFT are summarized in Table 1. As it can be seen, the E_HOMO_ energy values of **1**–**3** were greater than that of **4** (−5.905 eV), suggesting that the presence of a hydroxyl group could facilitate the interaction between the ligand and an appropriate receptor site. Furthermore, the LUMO orbital energy shows the same trend as the gap energy. In other words, **2** has more electron-accepting capability and **4** has the lowest reactivity, in agreement with the previously reported experimental results [23].

**Figure 4:**
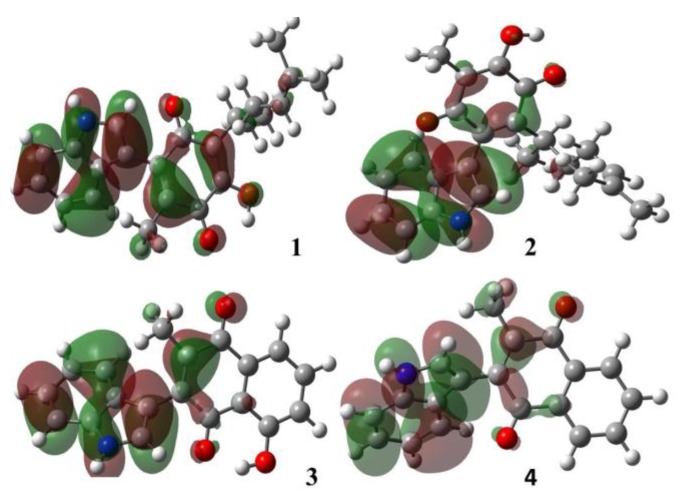
Molecular HOMO orbitals of the target molecules.

**Figure 5:**
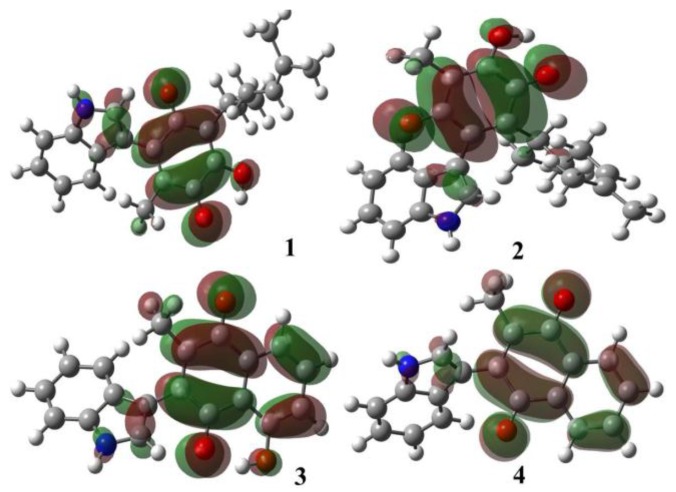
Molecular LUMO orbitals of the target molecules.

The contours of the frontier molecular orbitals for the studied molecules are displayed in Figure 4 and Figure 5. 

In all cases, the HOMO orbitals were located on the indole moiety and partially on the conjugated double bonds in the quinone motif; these π-electrons regions are available to perform electrophilic attack, in other words, these substrates behave as nucleophiles. The LUMO orbitals were located around the quinone ring; this fact is attributed both to the withdrawing-inductive and resonance effects of the conjugated carbonyl group allowing a nucleophilic behavior, in other words the substrate acts as an electrophile.

#### 2.1.2. Atomic Charges

**Figure 6:**
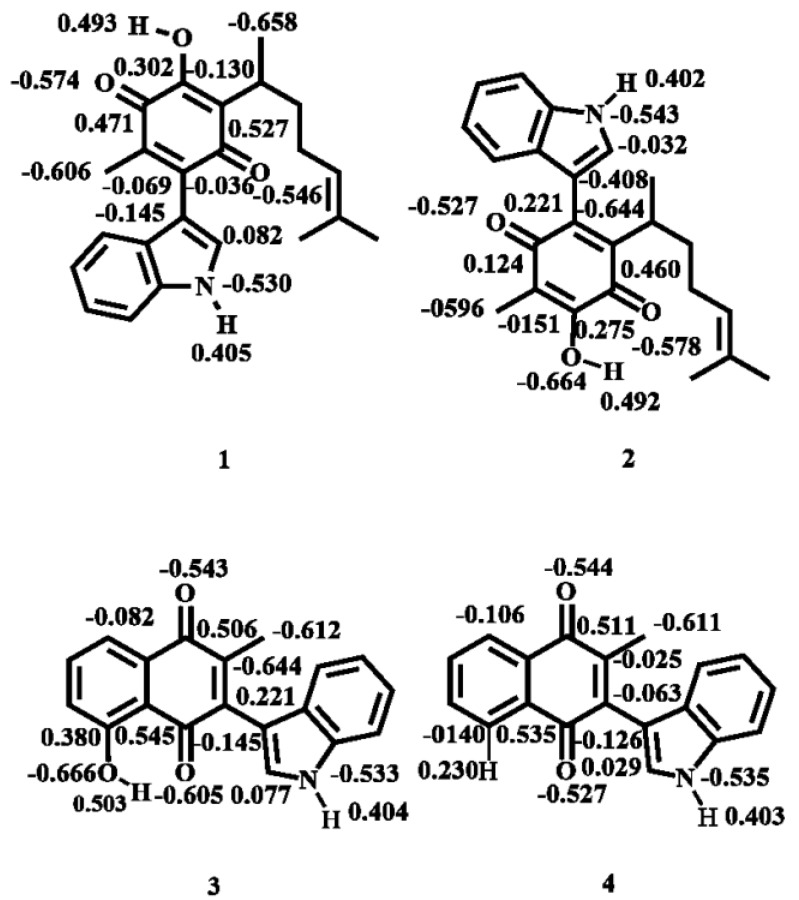
Charge distribution of selected atoms.

Charge values of the target molecules are displayed in Figure 6. As previously mentioned, the studied molecules presented cytotoxicity in cancer cells, an effect due to the corresponding interaction of **1**–**4** with receptor sites, mostly by non-covalent interactions between the ligand and amino acid residues of the protein receptor. In this sense, the natural population of charges was determined in order to identify possible interaction sites of the ligand molecules with the studied protein residues.

Analysis of the results shows that the most negative charge is localized on the oxygen atom of the hydroxyl group, consequently molecules **1**–**3** are the more reactive, a property suitably explained by a strong intramolecular hydrogen bond between the hydroxyl group with the adjacent carbonyl group (O-H···O = C). In addition, molecule **4**, which does not have a hydroxyl group, presents the most negative charge on C9, ascribed to the methyl group bonded to the quinone ring; this situation, can be extended to the second atom in order of negative charge for **3** (C9) **1** (C7) and **2** (C2), a behavior conveniently explained by the electron-donating resonance effect of the nitrogen atom present in the indole moiety. 

On the other hand, sites with high positive charge are localized at the carbons atoms of the closer carbonyl moiety, but not affected by the resonance effect of the indole group, and moreover not affected by intramolecular hydrogen bonding due to the hydroxyl group; as a complementary fact, the second atoms in order of positive charge correspond to the protons attached to the hydroxyl groups. In addition the respective positive charge values correspond to the hydrogen atoms joined to the nitrogen atom in the indole moiety. It is convenient to highlight that these hydrogens could be involved in hydrogen bonds with the receptor sites of proteins. 

#### 2.1.3. Molecular Electrostatic Potential Maps

The potential electrostatic map is an advantageous technique employed to both appropriately predict the molecular reactivity in addition to performing biological studies of compounds of interest [27]. In other works, they have been contemplated as an indicator of the reactivity regions of a target molecule, hence they have been employed in order to study electron-donator and electron-acceptor interactions, for example, between a drug and the amino acid residues of the cellular receptor [28].

**Figure 7:**
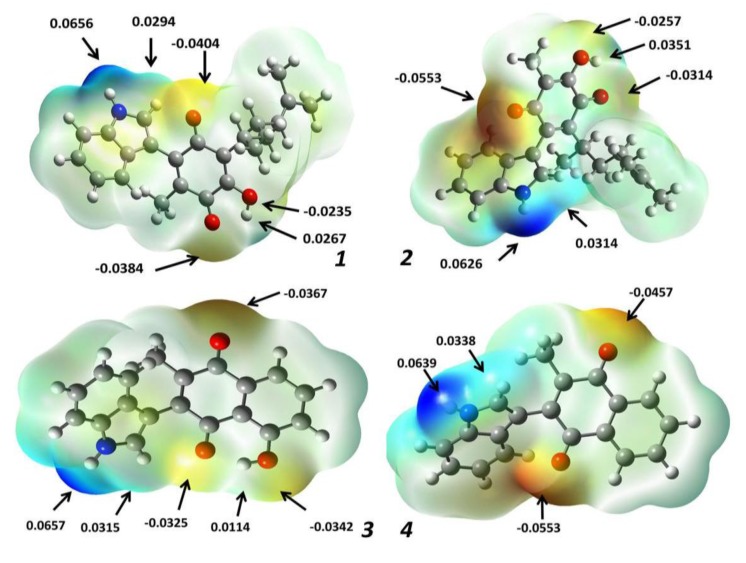
Molecular electrostatic potential surface of **1**–**4**.

Thus, this property was calculated for the four target molecules at the B3LYP/6-311++G (d,p) level of theory, and indicated by a color range from +6.6 × 10^−2^ (deepest blue) to −6.6 × 10^−2^ (deepest red) in the corresponding maps displayed in Figure 7, which show in red the nucleophilic sites, in blue color the electrophilic sites and the potential values are indicated in the highest electron density sites. It can be seen that the observed distributions evidence, in all cases, a bigger electronic density placed in the oxygen atoms of the carbonyl groups (values range of −0.0314 to −0.0553) and a minor distribution on the hydroxyl group oxygens (potential values between −0.0235 and −0.0342). This electrostatic potential decrease corresponding to the oxygen atoms of hydroxyl groups is a consequence of the intramolecular hydrogen bonds. In addition, the highest electronic deficiencies in the target molecules are located at H-1′ (+0.0626 to +0.0657), the hydrogen atom bonded at the indole moiety nitrogen. It is convenient to note the presence of another electron density deficiency at H-2′ bonded at the C_2′_ carbon atom in the indole moiety (+0.0294 to +0.0338). This is in addition to the hydrogen atom of the hydroxyl group (+0.0114 to +0.0351). Consequently, it is important to highlight that these atoms would participate in non-covalent interactions with some receptor amino acid residues.

### 2.2. Toxicological and Physicochemical Properties Prediction

The target molecules presented cytotoxic effects in human breast cancer cells, so a prediction of their toxicity risk and some significant physicochemical properties were performed using OSIRIS-Property-Explorer [29]. The corresponding results are summarized in Table 2.

The toxicity risk predictor shows that the compounds with less risk of undesirable effects are **3** and **4**, which do not present risks of mutagenicity, tumorigenicity, irritant or reproductive effects in comparison with **1** and **2** which present high risk of irritant effects. These results suggest that the risk is due to the presence of the aliphatic chain, explaining why the compounds **3** and **4** do not present this effect.

Physicochemical properties of the compounds were also estimated, such as cLogP, the logarithm of its partition coefficient between *n*-octanol and water, which is a property that describes the molecular hydrophobicity. The value range for this property varied from 2.83 to 4.6 in the studied compounds, with **3** and **4** presenting the lower values [30,31]. As the target molecules’ values are less than **5**, it indicates a reasonable probability they will be well absorbed [29,32]. Drug solubility (expressed as Log S) is an important factor to describe the absorption process. Poor solubility leads to poor absorption and bioavailability [30,31,32,33]. The common commercial drugs have values of Log S greater than −4. It is important to highlight that the best solubility is also observed for **1** and **2**; indicating that these compounds possess the best absorption, movement in the bloodstream and better elimination by the urinary tract. The compound with the lowest solubility was **4** which showed a low absorption value that could be the cause of the previously observed lower cytotoxic effect [23]. Regarding the molecular weight, all the compounds had values greater than 160 and less than 500 in agreement with Lipinski’s rule of five [33]. Furthermore, another good descriptor of the absorption is the total polar surface area (TPSA), including intestinal absorption, bioavailability, Caco-2 permeability and blood-brain barrier penetration [34]. The value of TPSA for the molecules **1** to **3** were the same (70.16), while in the case of **4** it was 49.93, confirming the lower absorption in comparison with the other studied molecules.

The drug score (DS) is the combination of drug likeness, cLogP, log S, molecular weight and toxicity risks into one handy value that could be used to judge a compound’s potential to qualify as a drug [35]. In this sense, **3** presented the best drug score while **1** and **2** had the lowest values. The lower drug score predicted for **1** and **2** was a consequence of the high probability of irritant effects in these molecules, however, **1** and **2** presented the best parameters in the physicochemical property prediction for drug use. 

### 2.3 Docking Study in the Apoptosis Pathways

**Figure 8:**
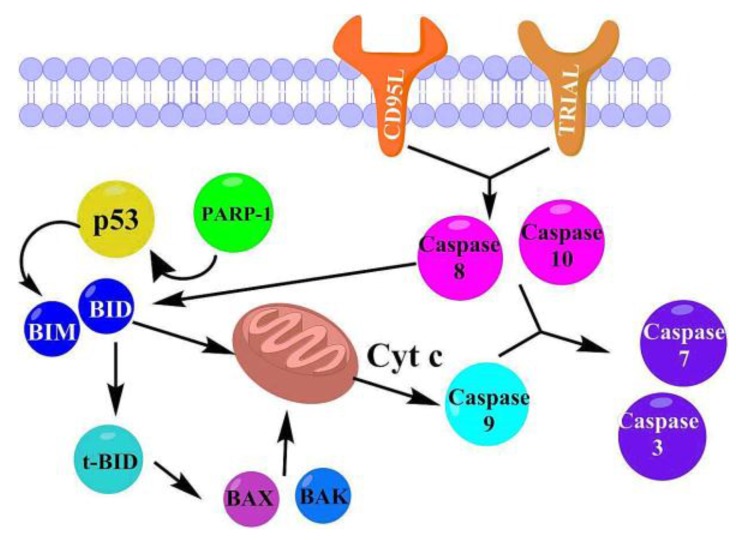
Intrinsic and extrinsic pathways of apoptosis.

It is convenient to recall that we have previously reported experimental cytotoxicity results for the studied compounds in cancer cells [23]; the morphology results of the treated cells suggested a possible apoptotic pathway. In addition perezone, isoperezone [13], plumbagin [36,37] and some other indolylquinones [38,39] have also shown apoptotic activity. It is additionally interesting to note that several quinones and naphthoquinones are recognized for their capability of association with certain proteins; the corresponding interaction can be assigned to a covalent-bond produced by a Michael type addition to the quinone ring. These molecules also can form other non-covalent interactions with specific proteins [40,41]. On the other hand, theoretical studies of the molecular docking of perezone [42] and plumbagin [43] with proteins involved in apoptosis have been previously performe. Accordingly, in this work, a molecular docking of the indolylquinones **1**–**4**, with the corresponding proteins involved in the intrinsic and extrinsic apoptosis pathways [44,45,46] was proposed (Figure 8), without considering any covalent binding; such a consideration must be taken into account due to the fact that the sites required to undergo a Michael interaction are already unavailable.

#### 2.3.1. Intrinsic Pathway Considerations

The first step in the considered activation mode of the intrinsic pathway was the interaction of the indolylquinones with poly(ADP-ribose) polymerase (PARP-1), activating p53 and then promoting the action of the BIM protein on the mitochondria. The mitochondrial stress causes both the release of cytochrome C, the conversion of the procaspase 9 in caspase 9, and finally the activation of caspases 3 and 7. Moreover, the molecular recognition among the ligands **1**–**4** with the proteins p53 and BIM, were also considered and then appropriately proved. On the other hand, when the ligand activates the intrinsic apoptotic pathway, without any interaction with p53, it is called the p53-independent route. Thus in this study, the p53-independent route is considered to involve the interaction between the target molecules directly with BIM protein.

#### 2.3.2. Extrinsic Pathway Considerations

The extrinsic pathway of the studied compounds interaction with the membrane receptors CD95L and TRIAL (Figure 7) was also studied. After the interaction with these receptors, the caspases 8 and 10 were activated. In addition, these enzymes can activate the caspases 7 and 3, or stimulate the conversion of BID in t-BID, promoting an interaction of t-BID with BAX or BAK and consequently producing mitochondrial stress. In this sense, the interactions of the indolylquinones with t-BID, BAX and BAK were also studied. The results of the molecular docking study with the aforementioned proteins are summarized in Table 3.

#### 2.3.3. Intrinsic Pathway Docking Study Results

Taking into account the intrinsic pathway, the molecules **1**–**4** need to penetrate the cellular membrane and act in the cytosol. Compound **1** and **2** have the best predicted penetration, considering their aforementioned predicted cLogP values (4.46 for both); meanwhile, **3** and **4** would have less penetration and minor activity in this route (cLogP = 2.81 and 3.17, respectively). In this sense, considering the last mentioned route, the first interaction would be with PARP-1, which does not have an interaction with **1**, this fact is also in agreement with the docking values of RMSD for this molecule ( RMSD = 22.569). The other three molecules have interactions with the PARP-1; being the stronger with **2** (score = −136.380), follow by **3** (score = −103.384) and finally **4** (score = −102.680). In this first step the order of interaction is in agreement with the experimental values, since compound **2** is the most active and **4** is the less active, in addition to the lower permeability predicted for **4**. 

**Figure 9:**
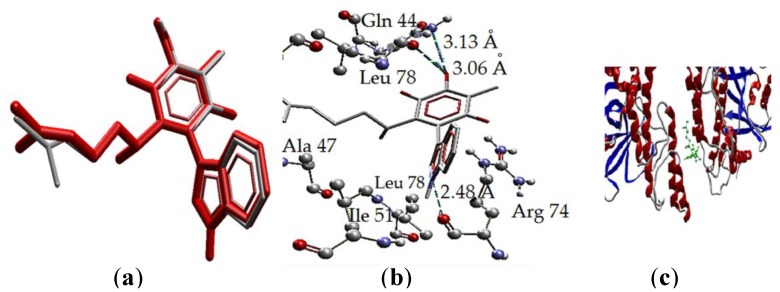
Interaction of **2** with PARP-1, PDB code (1UKO). (**a**) **2** and the pose, overlapping of the ligand (red) and the pose (grey). (**b**) Interaction of **2** and PARP-1 complex, three H bonds with protein residues. (**c**) Secondary structure, ligand (green) in the middle of the two isomers protein.

The interactions of **2** with the amino acid residues of PARP-1 are displayed in Figure 9. The binding of **2** with the protein is by three hydrogen bonds (H bonds), two of these bonds are between the hydroxyl group of isoperezone with Gln44 and Leu78, and the third is between the H1’ in the indole moiety and the Arg74 residue. The first hydrogen bond in the hydroxyl group had a distance of 3.13 Å where the amide group of the glutamine residue is the donor and the oxygen (the bigger negative charge) of the hydroxyl group is the acceptor. The second hydrogen bond is between the hydroxyl group as donor while the leucine carbonyl group is the acceptor and the distance between the atoms is 3.04 Å, being the biggest of the mentioned interactions. Finally, the stronger hydrogen bond is between the N–H group as donor, corresponding to the surface with high electronic density deficiency (in the MEP), and the carbonyl of the Arg46 residue with a distance of 2.48 Å (Figure 9b). Overlapping of the ligand (red) and the pose (grey) is shown in Figure 9b, with small differences in the aliphatic chain conformation. Moreover, the hydrogen bonding and arrangement of **2** in the protein (1UKO) secondary structure is exposed in Figure 9b,c, respectively.

On the other hand, the interaction of **3** with PARP-1 is formed by only one hydrogen bond. This bond is between the carbonyl group of Arg74 as acceptor and the hydrogen (high positive charge) of the hydroxyl group; the bond length is 3.08 Å, indicating a strong H bond and the hydrogen bond energy of −2.5 a.u., which is the maximum value for an H bond, in the Molegro software set. Finally, the docking of **4** does not present hydrogen bonds with PARP-1, for this reason the interaction energy value was the lowest of the four ligands.

**Figure 10:**
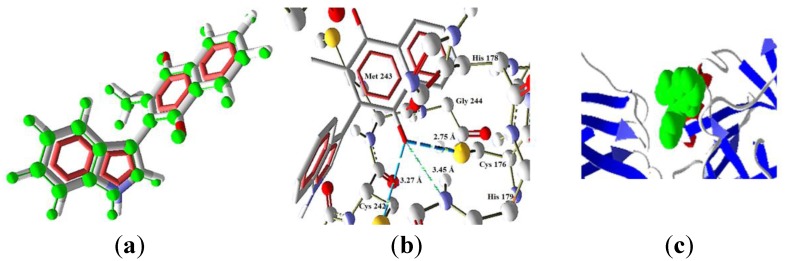
Interaction of **4** with p53. (**a**) **4** overlapping of the ligand (red) and the pose (grey). (**b**) Interaction of **4** and p53 (PDB code 2OCJ) complex, three H bonds with protein residues. (**c**) Secondary structure, ligand (green) in the middle of the two monomers.

The function of PARP-1 is to activate another protein, p53, which does not present any interaction with **1**–**3**. Molecule **4** shows values that indicate an interaction (Molscore = −97.960). This result indicates the possibility that **4** could activate the intrinsic pathway via p53. The docking of **4** with p53 showed excellent overlapping between the pose and the ligand conformation (Figure 10a). Additionally, three hydrogen bonds were found (Figure 10b); these bonds are between the oxygen atom of the carbonyl group O4 (bigger electronic density in the MEP) and the residues Cys176, Cys242 and Hys179, being the stronger bond the one with Cys176 (2.51 Å and bond energy of −1.72 a.u.). 

**Figure 11:**
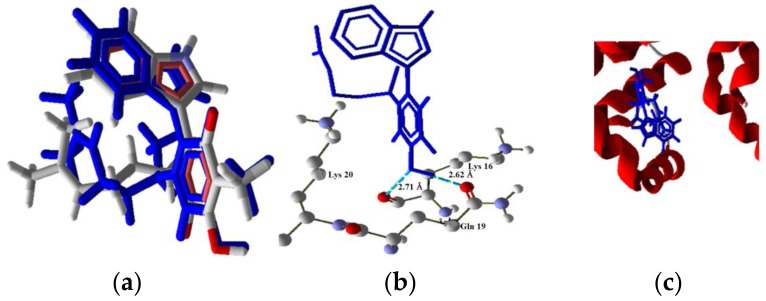
Model of interaction of **2** with Bim. (**a**) Poor overlapping of the ligand (blue) and the pose (red). (**b**) Interaction of **2** and Bim complex, two H bonds with protein residues. (**c**) Secondary structure, ligand (blue) in the middle of the two monomers.

The next proteins in the cascade are members of the family called “BH3-only” known as Bim. This protein is activated in the intrinsic pathway p53 dependent and independent. The results show the interaction of **2** and **4**, where the interaction energy decreases from **2** to **4** (scores of −141.566 and −100.855, respectively). These results continue the trend whereby **4** had the smallest interaction energy with the proteins involved and causes minor cytotoxicity. Furthermore, the compounds **1** and **3** did not show any molecular docking with this protein (RMSD values of 6.99 and 2.744, respectively). The interaction model (Figure 11) of **2** (high cytotoxicity) with the protein Bim was modeled with two hydrogen bonds between the hydroxyl group of **2** and the residues Lys16 and Gln19 at distances of 2.67 Å and 2.71 Å, respectively. This protein is important because the intrinsic pathway could be initiated in this point, independently from p53. Furthermore, the pathway could be activated by the four ligands. 

#### 2.3.4. Extrinsic Pathway Docking Study

The extrinsic pathway (Figure 7) was analyzed by interaction of the target molecules with the membrane receptors such as CD95L (4MSV) and TRAIL-R2 in two isoforms (4I9X and 4N90). Concerning CD95L, the molecules **1**–**4** have good docking results with these proteins, showing the best interaction with **2** (score = −132.437), followed by **4** (score = −105.411) and a lesser interaction with **1** (score = −99.340). 

**Figure 12:**
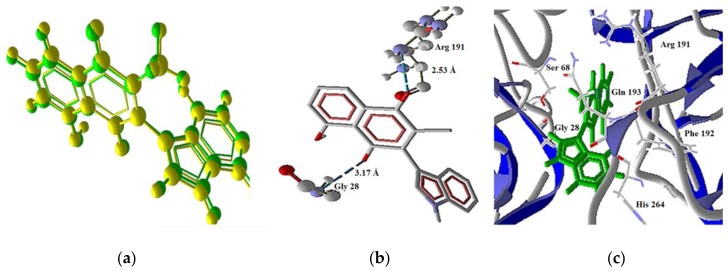
Model of the interaction of **3** with TRIAL-2 (4N90 isoform). (**a**) Compound **3** and TRIAL-2 complex, overlapping of the ligand (green) and the pose (yellow); (**b**) Interaction of **3** and TRIAL-2 complex, showing three H bonds with protein residues; (**c**) Secondary structure, ligand (green) in the middle of the two protein isomers.

Regarding the TRAIL-R2 isoforms, the 419X protein displayed docking only with **1** (score = −126.081 and RMSD = 1.342). However, the 4N90 protein interacts with **1** (score = −140.412 and RMSD = 1.677) and **3** (score = −121.757 and RMSD = 0.136). Molecule **3** establishes two hydrogen bonds, the first one between Arg191 as donor, and the oxygen atom (O1) of the carbonyl group as the acceptor; meanwhile a second kind of bond appeared between the Gly28 residue as donor, and the oxygen atom (O4) as acceptor (Figure 12). These observations are in agreement with the quantum calculation results, since the acceptors of the hydrogen are the two atoms with bigger negative charge and electronic density. A model of the interaction of **3** with 4N90 is shown in Figure 12.

The activation of these membrane receptors changes the procaspases 8 and 10 to caspases, which could active the caspases 3 and 7. Then, these proteins could promote the conversion of Bid to the t-Bid. The outcome showed molecular docking of **1**, **3**, **4** with Bid protein, where **1** had the best score of −157.069 and exhibits four hydrogen bonds with the quinone hydroxyl group as donor. The acceptors of the hydrogen bonds are: (a) the amine group of the His91 residue (2.77 Å, energy of −1.33 a.u.), (b) the oxygen atom of the Ser119 residue (3.175 Å, energy −2.157 a.u.), (c) the amine group of the Asp4 residue (3.023 Å, −2.5 a.u.) and finally the NH group of Met3 (2.732 Å, −2.07 a.u.). In this sense, the ligand **3** displays the lower interaction. The binding of the molecules with Bid could inhibit the protein-protein interaction that is an essential phenomenon for its function in the apoptotic pathway [46]. This effect is also observed with calestrol, a molecule with a similar moiety as the target molecules [47]; in this case the activity of caspase 8 on Bid in the extrinsic route can be inhibited. This effect explains the difference in cytotoxicity between **2** (RMSD of 52.699) and the rest of the studied molecules; additionally, there is the lower cytotoxicity of **1** as result of the high interaction with this protein (score of −157.069) in comparison with **3** (score = −141.290). 

**Figure 13:**
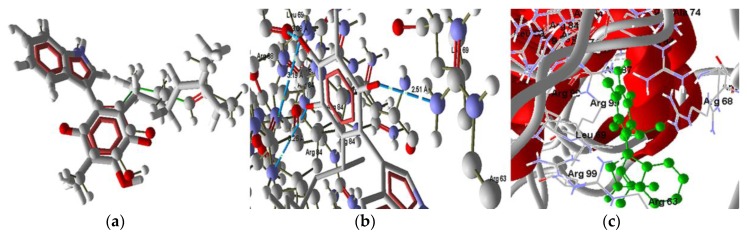
Model of interaction of **2** with t-BiID. (**a**) Compound **2** and t-BiID complex, overlapping of the ligand (red) and the pose (grey); (**b**) Interaction of **2** and t-BiID complex, showing three H bonds between the carbonyl group and the protein residues; (**c**) Secondary structure, ligand (green) in the middle of the protein.

The molecules **2**–**4** could interact with the protein t-Bid, being the stronger interaction with **2** (score = −1790.830). The binding mode of **2** with t-Bid is established by five hydrogen bonds (Figure 13), three of them with the oxygen atom (O4) as acceptor (high electronic density region in the MEP) and the Glu59 residue in three of the isomeric chains of the protein as donors. Moreover, there are two more bonds with the indole N–H group as donor (high electronic density difference in the MEP value) with the two Glu54 residues of isomeric chains as acceptors. The next molecule in order of affinity with this protein was ligand **4** (score = −1556.770), while ligand **3** has the lower interaction energy values. 

Finally, the activation of t-Bid causes the interaction with BAX (1FI6) y/o BAK (2IMT) which stimulates the release of cytochrome c. In this point, the target molecules **2**–**4** interact with BAX in order to strengthen the release. Compound **2** (score = −138.366) has the bigger interaction followed by **3** (score = −130.975). The interaction of **2** with this protein was proposed to occur by the formation of three hydrogen bonds with Asp2 (3.15 Å, H bond energy = −2.27 a.u.), Arg9 (3.09 Å, H bond energy = −2.5 a.u.) and Met1 (3.24 Å, H bond energy = −0.98 a.u.). In the case of the molecular docking with BAK, **2** has also the stronger interaction (score = −135.800) followed by **3** (score = −121.235) and the last is **1** (score = −116.52). Molecule **4** did not show docking with this protein (RMSD value of 2.179). 

#### 2.3.5. Proposed Apoptosis Pathways

Taking into account the previously described results, the proposed pathways are:

a) Molecule **2** acts by initiating the p53-dependent way, considering the interaction with PARP-1; this protein have an eventful contribution in important biological process as transcription and the regulation cellular cycle, DNA damage response, apoptosis and the preserver of genome integrity [48]. This activation induces proapoptotic BIM proteins, which stimulate BAX/BAK proteins, generating mitochondrial stress and promoting the intrinsic apoptotic pathway. Moreover, the p53-independent intrinsic pathway could be activated as consequence of the interaction of **2** with BIM protein. Ligand **2** has the bigger predicted permeability among the target molecules, though the extrinsic apoptosis pathways also can be initiated by recognition with FasL (CD95L) death receptor provoking the cellular death mediated by caspase 8. The high cytotoxicity in breast cancer cells was explained by the activation of the two pathways and the high interactions with the proteins involved in these routes. 

b) The next molecule in order to cytotoxic activity value was **3**, which also can activate the intrinsic (p53-dependent and -independent) and extrinsic pathway by recognition with CD95 and TRIAL membrane receptor (FasL receptors). However, this molecule displayed the lowest predicted permeability, making the most probable the extrinsic pathway and the lower interaction with BID (it has the less probability to inhibit the interaction of BID with caspase 8). Besides, **3** presented moderate interactions with the proteins studied. 

c) Molecule **1** can interact with the FasL (CD95L and TRAIL-R2) receptors, however it presented the biggest value of permeability and has high affinity for BID protein, so this molecule can inhibit the interaction among the caspase 8 and BID and suppress the extrinsic apoptotic pathway. Since it does not have a molecular docking conformation with PARP-1, the prospective pathway inside of the cell (considering the big predicted membrane permeability) is the activation of the proteins t-Bid and BAK (considering the moderate affinity in comparison with the other target molecules). 

d) Finally, the proposed pathway for **4** is the activation of the intrinsic (p53-dependent and -independent) and the extrinsic pathway for CD95L recognition; however the lower cytotoxic activity is a consequence of the low affinity with the proteins involved in the apoptosis, in addition of the low reactivity of this molecule.

### 2.4. Pharmacological and Metabolic Properties

#### 2.4.1. Target Molecules Absorption Predictions

Complementary and interesting results, considering the potential use of the studied molecules as drug, are the prediction of its absorption, metabolic and excretion properties of the studied molecules in humans. 

The behavior in different absorption and excretion models were predicted using admetSAR methodology and the results are given in Table 4. The predictions for **1**–**4** to exhibit human intestinal absorption have a probability value of 1 in each case. Concerning the blood brain barrier, the compounds **1**, **2** and **4** show medium absorption probability values, being ligand **4** the one displaying the biggest probability (0.872). 

These results are in agreement with the results obtained with OSIRIS, which indicated the lower water solubility of **4** (Log S value of −4.46); in addition, **4** is the less polar compound, since the structure does not have a hydroxyl group. A model considered the “gold standard” for the drug permeability is the human colon adenocarcinoma (Caco-2) monolayer cell culture which is widely used in drug discovery and has been recommended by the Food and Drug Administration (FDA) [49,50], in this sense the prediction for the target molecules show permeability in all cases. Regarding the P-glycoproteins, this molecule is one of the mayor ABD transporters, distributed in several tissues, and involved in the clearance of xenotoxins against steep concentration gradients, at the expense of ATP hydrolysis. Additionally, the P-glycoprotein plays an important role in the transport of small molecules in vital areas. In addition, this protein is present in the multidrug resistance cancer cells and its inhibition can be used to overcome the multidrug resistance [51,52,53]. In this sense, the molecules **1**–**3** showed possibilities of being good substrates when used with those proteins.

In respect to ligand **4**, which was a non-substrate, its bioavailability could be minor in comparison with the other studied molecules limiting its biological activity. This result is in agreement with the less activity shown in cancer cells. The non-substrate behavior of **4** is related with the fulfillment of the “rule of four”, which is assigned to compounds with less than 400 Da molecular weight, no more than four nitrogen and/or oxygen atoms and base pKa minor to 8 as non-substrate [54]. The prediction of the inhibitor character of indolylquinones studied in P-glycoprotein-I and II indicated that **1** and **2** are inhibitors of these proteins, while **4** only act as inhibitor in glycoprotein-I. In the case of **3** did not present inhibitory characteristics against the P-glycoproteins. Finally, none of the molecules considered present inhibitory effects on the renal organic cation transporter.

#### 2.4.2. Target Molecule Metabolism

Another interesting result was to predict the metabolism of the studied compounds using admetSAR and MetaPrint2D-React methodology (Table 5). Firstly, the behavior as substrate or inhibitor of the target molecules in the most important isoforms of the cytochrome P450 participants in the metabolism of therapeutic drugs was evaluated. During the biotransformation of the drugs, the molecules are broken down and/or converted into more soluble molecules, which play important roles in the pharmacokinetic and therapeutic action of drug molecules.

Evaluation of the target molecules as substrates of CYP shows that **1**–**4** act as non-substrates of the 2C9 and 2D6 isoforms of cytochrome P450 (CYP). The obtained results for these cytochromes can be explained by considering the usual structure of their substrates; CYP2C9 substrates possess weakly acidic properties and multiple aromatic rings [55]. The molecules **1**, **2**, **3** present a weakly acid hydroxyl group, however do not present a multiple aromatic ring, while **4** does not present any of these characteristics. Regarding CYP 2D6, the primary characteristic of their substrates is the presence of basic nitrogen atom placed 5 Å or 7 Å from the site of oxidation [56,57]. The structures of **1**–**4** have a nitrogen atom in the indole motif, however, the unshared electron pair is not available due to the resonance effect, and also the distance between the nitrogen and the aromatic group in **3** and **4** or the double bond C14–C15 in **1** and **2** are placed in a distance different from 5 Å or 7 Å. The substrate behavior prediction in the CYP3A4 indicated that only **1** and **2** could be substrates, because these molecules meet the characteristics of the pharmacophore model for this cytochrome. The molecules **1** and **2** present, by considering the MEP and charge, two hydrogen bond acceptors (O1 and O4), one hydrogen bond donor (OH–3, OH–6 or N–H) and one hydrophobic region (aliphatic chain for C8 to C15), (*vide supra*). Additionally, an important metabolic property to consider is the capability of these compounds to inhibit the CYP isoforms, which is an adverse effect.

Prediction of the ligand effect in CYP1A2 show the inhibitory effect of **1**–**4**; this result could be explained considering the similitude between the substrates (polar heterocyclic compounds and arylamines) of cytochrome 1A2 [56,57,58,59] with the indole motif of the target molecules. Another CYP isoform inhibited by the studied molecules is the –CYP2C9, in agreement with result obtained in the substrate behavior studies. In the same way, **3** and **4** show inhibitory effects on CYP2C19. On the other hand, the compounds **1**–**4** did not show inhibitory effect on CYP2D6 and CYP3A4. 

#### 2.4.3. Phase I and II Metabolic Pathway

**Figure 14:**
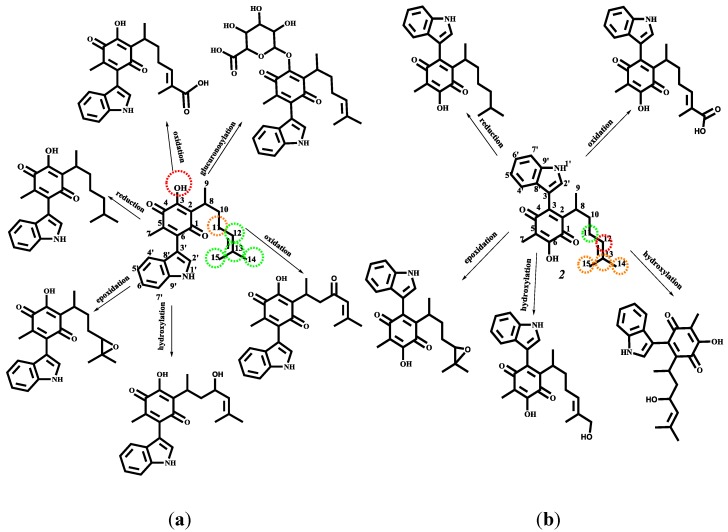
Predicted metabolism for: (**a**) indolylperezone and (**b**) indolylisoperezone.

Finally, the study of the possible way to degradation of **1**–**4** during the human phase I and II metabolism was predicted using the MetaPrint2D-React program [60,61]. The results are displayed in Figure 12 for **1** and **2**. Meanwhile, the metabolism pathway of **3** and **4** is shown in Figure 14. 

**Figure 15:**
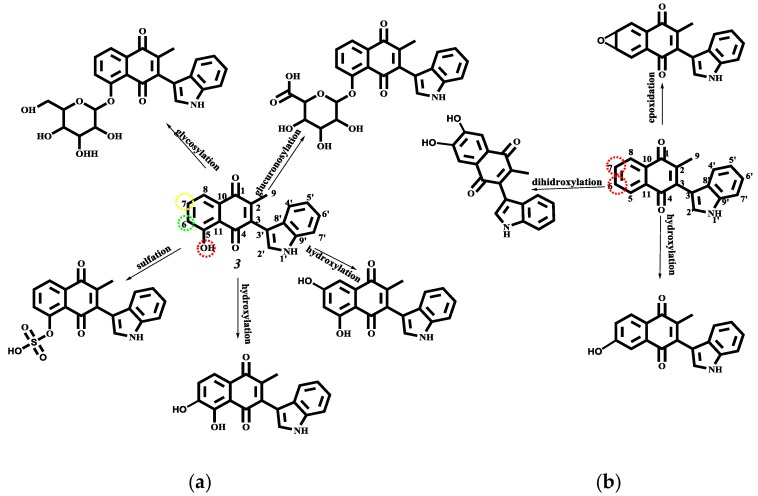
Predicted metabolism for: (**a**) indolylplumbagine (**3**) and (**b**) indolylmenadione (**4**).

Molecule **4** corresponds is the least reactive compound, and this is reflected in the number of metabolites predicted. The color highlighting an atom indicates its normalized occurrence ratio (NOR), Figure 14 and Figure 15. A high NOR indicates a more frequently reported site or metabolism in the metabolite database of the program. The red color indicates high values of NOR (from 0.66 to 1), the orange color designate values between 0.33 to 0.66, the green color indicates a NOR value range of 0.15 to 0.33, white color, range values of 0.00 to 0.15 and grey, denotes lack of data. The high NOR in **1** is exhibited in the hydroxyl group, indicating a possible glucoronosylation increasing the solubility and the excretion of the compound. The C–11 position, present the next NOR value proposing a hydroxylation and oxidation on this carbon, producing an allylic alcohol or an α,β-unsaturated ketone. Additionally, the transformation of the double bond and the oxidation of the carbons C14–C15 has the lowest NOR value. The possible metabolites produced are the epoxide at C12–C13, the C12–C13 reduction product and the product of oxidation to a carboxylic acid at C14–C15. Compound **2** reveals similar metabolites in comparison with **1** However, the glucoronosylation and the oxidation to ketone in C–11 are not shown. In contrast the allylic hydroxylation in C14 or C15 is predicted.

Concerning the metabolism of **3** and **4**, since the first has a hydroxyl group (a group with high NOR value), this molecule can be conjugated with glucoronic acid or some glycosides to increase the solubility and the compound excretion; moreover, the sulfation of the hydroxyl is also predicted. The metabolism reaction on **3** and **4** aromatic systems are the hydroxylation via production of the corresponding dihydroxylated compounds; additionally in **4** an epoxide at C6–C7 is proposed.

## 3. Methods

### 3.1. Reactivity Parameters

Theoretical calculations were carried out using the corresponding structures previously optimized of the target molecules [23] by Density Functional Theory (DFT) [62] with GAUSSIAN09 program [63], employing Becke’s three parameters exchange and the Lee-Yang-Parr correlation hybrid functional (B3LYP) [64] with the 6–311++G(d,p) basis set, including the split-valance and of diffuse functions [65,66,67,68]. The highest occupied molecular orbital-lowest unoccupied molecular orbital (HOMO-LUMO) gap is a typical quantity used to describe the dynamic stability of molecules [30,69]. Values of the orbital energy and the surface of the frontier orbitals were calculated using the same level of theory. Natural Bond Orbital (NBO) was used for electron natural population analysis in Gaussian program. Natural Population Analysis was used for comparing differences rather than determining absolute atomic charges. The charge analysis was performed to explain the model of interaction of the corresponding ligand with the probed proteins [70,71]. Molecular electrostatic potential maps (MEPs) were obtained for the target molecules to complete the electronic analysis considering the importance of these results in the interaction models in the studied biological system [28].

### 3.2 Toxicological and Physicochemical Properties Prediction

Physicochemical properties predicted for the molecules under study provide pertinent information about the facility of a drug molecule to interact with the amino acid residues inside cells or the membrane receptors. The toxicological risk and physicochemical properties of the studied molecules were obtained using the OSIRIS property explorer. The toxicological risk prediction process relies on a precompiled set of structural fragments that give rise to toxicity alerts in case they are encountered in the structure currently drawn. LogP and log S were estimated by using the OSIRIS method, which is implemented as system for adding the contributions of every atom based on its properties. The drug like-ness approach is based on a list of about 5300 distinct substructure fragments with associated drug-likeness scores. The drug likeness is calculated employing the score values of those fragments present in the molecule under investigation [29,32,36].

### 3.3 Docking Study

#### 3.3.1 Docking Details

Molecular docking predicts the binding capability of a small molecule to a target protein by developing a relatively stable complex. The predicted ligand orientations allow the prediction of the preferred binding conformations and affinity between the proteins and small molecules as ligands [25]. The small molecules with high-binding affinities are identified as potential candidates for follow-up experimental validation. Traditionally, molecular docking is primarily employed to screen pure compounds; the top-ranking chemicals are subsequently selected for experimental validation. Unlike synthetic chemicals, most natural products are commercially unavailable, although several similar or analogue products could be obtained. Besides, most of the three dimensional protein structure is ready obtained from the Protein Data Bank, by using the appropriate code.

#### 3.3.2. Molecular Docking Simulation

Molecular docking simulations were performed using the three dimensional crystal structures of ten proteins from the PDB dataset: Parp1 (1UKO); BAX (1FI6); BID (2BID); BAK (2IMT); tBID (2M51); p53 (2OCJ), BIM (4I9X); TRAIL-R2(4N90 and 4YK9); and CD95L (4MSV), respectively, from the RCSB, Protein Data Bank [72,73,74,75,76,77,78,79,80,81,82]. The Molegro software package [83] was used for all docking conformations and analysis. The docking parameters for Molegro docking study were maintained at their default values. The ligand was not rigid and allowed to torsion in the first blind docking processes. The grid box was 15 Å × 15 Å × 15 Å, encompassing the ligand binding cavity of each protein. Only a maximum of five surfaces for docking were fixed for the typical docking process. The binding modes were clustered using the root-mean-square deviation among the cartesian coordinates of the ligand atoms. The docking results were ranked according to the binding free energy and the root mean standard deviation RMSD < 2 Å) (values of the corresponding ligand protein complex, in order to compare with the leader drug. The binding modes with the more negative binding free energy (Docking score) and maximum hydrogen bonding number were selected as the optimum docking conformation. The binding results are graphically presented using the PyMOL Molecular Graphics System Version 1.3 [84] and the Molegro visualizer.

### 3.4. Pharmacological and Metabolic Properties

Absorption and metabolic properties of the studied compounds were calculated by using the admetSAR server [49,85], which predict about 50 ADMET endpoints using a chemo- informatics-based toolbox, called ADMET-Simulator, which integrates high quality and predictive QSAR models. The proposed human metabolism for the target compounds was made using MetaPrint2D-Reaction software, a prediction set of xenobiotic metabolism by means of data-mining and statistical analysis of known metabolic transformations reported in scientific literature [86,87,88,89,90,91]. The results are displayed in circular colored marks predicting the reactions at that site and the possible reaction types, showing the metabolite formed. The color of the mark in the atoms indicates its normalized occurrence ratio (NOR) values. High NOR value indicates a more frequently reported site of metabolism in the metabolite database.

## 4. Conclusions

An in silico study of the apoptosis pathway for a set of several novel indolylquinones was carried out, being important to note that the obtained theoretical results are in agreement with the data reported in an experimental cytotoxic study previously performed by our research group.

The most cytotoxic compound was **2**, with the smallest energy gap of the HOMO and LUMO orbitals and consequently big reactivity values; meanwhile, the less cytotoxic compound **4**, showed the least reactivity and the biggest energy gap value. These results are indicative of a possible correlation between the chemical stability and breast cancer cell cytotoxicity. In addition, the toxicity risk predictor shows that the compounds with less risk of undesirable effects are molecules **3** and **4**, without any risks of mutagenicity, tumorigenicity, irritant or reproductive effects, while molecules **1** and **2** show a high risk of irritant effects. These results indicate that the risk is due to the presence of the aliphatic chain, therefore the compounds 3 and 4 do not present this effect. Moreover, the respective docking studies of the target molecules as ligands, considering the different proteins involved in the different pathways were also carried out; in this sense, a data analysis related to the anticancer mechanism of action was conveniently performed. The results are in agreement with a previously experimental study of the ligands’ cytotoxicity. Feasible apoptotic pathways and explanation of the corresponding IC_50_ values by each molecule were also achieved.

On the other hand molecules **1**–**3** were the more reactive since the most negative charge is localized on the oxygen atom of the hydroxyl group of the studied molecules. Furthermore, molecule **2** could be interacting with the receptor site showing its pharmacological activity; consequently, molecule **4**, which does not have a hydroxyl group, is the less reactive. The biggest electronic density was observed on the oxygen atom of the carbonyl groups, and the minor value on the oxygen of the hydroxyl group. In addition, the most electronic deficiency in the target molecules were observed in H–1’ hydrogen atom, bonded at the nitrogen in the indole moiety, followed by H–2’ bonded at the carbon atom in the indole moiety, and the hydrogen atom of the hydroxyl group. Consequently, it is important to highlight that these atoms could participate in non-covalent interactions with some receptor amino acid residues.

In regards to several physicochemical properties (cLogP, log S, molecular weight and toxicity risks) that were obtained for the target compounds, it must be noted that were estimated using bioinformatics in order to evaluate the compounds as possible drugs; thus, compound **3** offered the best drug score while the molecules **1** and **2** presented the worst values. In addition, the less drug score predicted for compounds **1** and **2** was considered a consequence of the high probability of irritant effects in these molecules. However, **1**–**2** gave the best parameters in the physicochemical properties prediction for a drug use.

Absorption and excretion models indicate that the exhibited human intestinal absorption for **1**–**4** have a probability of 1. Concerning the blood brain barrier, the compounds **1**, **2** and **4** showed medium absorption probability values. In respect to ligand **4** (a non-substrate), its bioavailability could be minor in comparison with the other studied molecules, limiting its biological activity. This result is in agreement with its lower activity value shown in cancer cells. Lastly, none of the molecules considered present inhibitory effects on the renal organic cation transporter.

Finally, the possible degradation pathways of **1**–**4** during human phase I and II metabolism were predicted. Molecule **4** corresponds to the less reactive compound, reflected in the number of predicted metabolites.

## Figures and Tables

**Table 1:** E_HOMO_ and E_LUMO_ of the studied molecules.

Molecule	Energy (eV)	
	HOMO	LUMO
**1**	−5986	−3374
**2**	−5959	−3428
**3**	−6013	−3347
**4**	−5905	−3184

**Table 2:** OSIRIS toxicological and physicochemical predicted properties.

Property		Compound			
		1	2	3	4
Toxicity Risks	Mutagenic	N	N	N	N
	Tumorigenic	N	N	N	N
	Irritant	H	H	N	N
	Reproductive effect	N	N	N	N
Physicochemical Properties	cLogP	4.6	4.6	2.83	3.17
	Solubility (Log S)	−4.07	−4.07	−4.15	−4.46
	Mol. weight	363	363	303	287
	TPSA	70.16	70.16	70.16	49.93
	Druglikeness	−0.87	−0.87	2.29	−0.57
	Drug Score	0.24	0.24	0.74	0.5

**1**: indolylperezone, **2**: indolylisoperezone, **3**: indolylplumbagin, **4**: indolylmenadione; N = no risk, H = High risk.

**Table 3:** Energy values of the interaction (score) between the ligands with the protein and their root mean standard deviation (RMSD in Å).

Protein (PDB)	Target Molecules							
	1		2		3		4	
	Score	RMSD	Score	RMSD	Score	RMSD	Score	RMSD
PARP-1 (1UKO)	−144.263	22.569	−136.380	0.702	−103.384	0.117	−102.680	0.254
p53 (2OCJ)	−115.059	5.92	−102.806	2.274	−107.622	1.975	−97.960	0.212
BID (2BID)	−157.069	0.654	−133.750	52.699	−141.290	0.082	−137.550	0.16
BIM (4YK9)	−126.480	6.99	−141.566	0.837	−99.742	2.744	−100.855	0.142
CD95L (4MSV)	−99.340	0.6789	−132.437	0.457	−102.592	0.17	−105.411	0.127
TRAIL-R2 (4I9X) (4N90)	−126.081	1.342	−137.349	16.801	−101.123	6.465	−101.083	7.82
	−140.412	1.6765	−132.437	136.013	−121.757	0.136	−122.564	31.917
t-BiID (2M51)	No docking	No docking	−1790.830	0.454	−1552.800	0.073	−1556.770	0.389
BAX (1FI6)	−140.360	9.819	−138.366	0.884	−130.975	0.08	−126.530	0.072
BAK (2IMT)	−116.520	0.3111	−135.800	0.868	−121.235	0.146	−95.060	2.179

**Table 4:** Prediction of the absorption of the target molecules in different models.

Absorption Model	Molecules							
	1		2		3		4	
	Result	P	Result	P	Result	P	Result	P
Blood-Brain Barrier	BBB+	0.650	BBB+	0.556	BBB−	0.570	BBB+	0.872
Human Intestinal Absorption	HIA+	1	HIA+	1	HIA+	1	HIA+	1
Caco-2Permeability	Caco2+	0.500	Caco2+	0.507	Caco2+	0.527	Caco2+	0.646
P-glycoprotein Substrate	S	0.722	S	0.692	S	0.620	NS	0.511
P-glycoprotein Inhibitor	I	0.536	I	0.604	NI	0.818	I	0.742
	I	0.946	I	0.946	NI	0.897	NI	0.711
Renal Organic Cation Transporter	NI	0.769	NI	0.793	NI	0.834	NI	0.746

**1**: indolylperezone, **2**: indolylisoperezone, **3**: indolylplumbagin, **4**: indolylmenadione; + = positive to absorption, − = negative to absorption, S = substrate, I = inhibitor, NI = non-inhibitor NS = non-substrate, P = probability.

**Table 5:** Prediction of metabolism.

Metabolism Model	Molecules							
	1		2		3		4	
	Result	P	Result	P	Result	P	Result	P
CYP450 2C9 Substrate	NS	0.764	NS	0.794	NS	0.740	NS	0.734
CYP450 2D6 Substrate	NS	0.810	NS	0.807	NS	0.818	NS	0.814
CYP450 3A4 Substrate	S	0.678	S	0.646	NS	0.569	NS	0.500
CYP450 1A2 Inhibitor	I	0.637	I	0.664	I	0.912	I	0.914
CYP450 2C9 Inhibitor	I	0.510	NI	0.511	I	0.907	I	0.894
CYP450 2D6 Inhibitor	NI	0.812	NI	0.812	NI	0.687	NI	0.530
CYP450 2C19 Inhibitor	NI	0.589	NI	0.619	I	0.719	I	0.840
CYP450 3A4 Inhibitor	NI	0.809	NI	0.808	NI	0.830	NI	0.707

**1**: indolylperezone, **2**: indolylisoperezone, **3**: indolylplumbagin, **4**: indolylmenadione; S = substrate, I = inhibitor, NI = non-inhibitor, HI = high inhibition, NS = non-substrate, P = probability.
